# Fused Filament Fabrication Printer Modifed to Dispense Cement Paste for Concrete Additive Manufacturing Studies

**DOI:** 10.6028/jres.125.034

**Published:** 2020-11-24

**Authors:** Scott Z. Jones

**Affiliations:** 1 National Institute of Standards and Technology, Gaithersburg, MD 20899, USA

**Keywords:** automated construction by extrusion, cement paste 3-D printer, concrete 3-D printing

## Abstract

Additive manufacturing (AM) with cement-based materials is an emerging technology that has the potential to revolutionize concrete construction. The placement process is quite complex, requiring suffcient flow properties as the material leaves the nozzle, and suffcient stiffening properties before the subsequent layer is placed. Precise control of material proportions and in-line monitoring of the time-dependent rheology are required to ensure the successful adoption of AM in the concrete construction community. To facilitate the study of the rheological properties of cementitious materials, as they pertain to AM, a commercial bench-top fused filament fabrication three-dimensional (3-D) printer was modifed to dispense cement paste mixtures. Modifcations included the design and assembly of a pumping system and software modifcations to the 3-D printer’s firmware that were necessary to accommodate the new hardware. After assembly, a series of tests were conducted to verify machine movements and to calibrate the number of step pulses required per unit volume of extruded paste. The resulting software modifications and configuration files are publicly available

## 1 Introduction

Additive manufacturing with cementitious materials, such as concrete, has been studied since 1995 with the invention of the large-scale automated construction technology ContourCrafting^1^1Certain commercial equipment, instruments, or materials are identified in this paper to foster understanding. Such identification does not imply recommendation or endorsement by the National Institute of Standards and Technology, nor does it imply that the materials or equipment identified are necessarily the best available for the purpose. [1]. Since that time, the amount of research on this topic has increased, with more groups beginning projects on what has become to be known as additive construction by extrusion (ACE) or three-dimensional (3-D) concrete printing (3DCON). The ACE process is characterized by extrusion of a cementitious material through a nozzle and subsequent deposition, in a layer-by-layer fashion, by a computer-controlled robot. This technology falls within the digital fabrication category, a broader category of construction technologies that utilize automation to fabricate components [2]. Smart Dynamic Casting (SDC) is one such technique. Here, the material's final state is shaped by automatic control of a form described in Refs. [3] and [4]. An alternative to ACE is particle bead 3-D printing, where the final shape of the object is realized by bonding particles in a packed bed together using an adhesive fluid as described in Refs. [5-7].

The ACE process has the potential to revolutionize concrete construction by reducing the cost of formwork assembly and by enabling real-time quality control measurements of the material. Before the construction industry can fully adopt ACE, several material science challenges must be solved and practical solutions implemented. Buswell et. al., [8] described several of the challenges to implementing ACE and classifies them into three technical areas: fresh state of the mixture, hardened properties, and geometric conformity. The fresh properties of the mixture and their relationship to printing properties, such as geometric conformity, remain difficult to study because a commercially available apparatus with which to test the automated placement of cementitious material does not exist.

In this paper, a fused filament fabrication (FFF) 3-D printer intended for thermoplastic polymeric materials was modified to dispense a cement paste mixture to address this research equipment gap. Hardware modifications included the design of a pumping and nozzle system and an external control system to monitor printing parameters and control portions of the pump. Modifications to the 3-D printer's firmware were necessary to accept the newly designed hardware and maintain temperature compatibility with cement paste. Once complete, the pump extrusion rate was calibrated using National Institute of Standards and Technology (NIST) SRM 2492 [9], which is a dense suspension with Bingham flow properties similar to cement pastes. Once the flow rate and the lateral nozzle movements were calibrated, a MATLAB script was used to generate the G-code commands necessary to build a tall, thin structure.

A detailed overview of the construction and operation of a cement paste 3-D printer, located in the Engineering Laboratory of NIST, is given so that others may re-create the capabilities for their purposes. An overview of FFF is given, followed by a description of the pumping and nozzle system, control of the 3-D printer, and operation of the 3-D printing to test cement paste. Parts and assembly details are provided to the level necessary to assemble the instrument, and the computer code necessary for the specific differences associated with extruding cement paste instead of plastic will be made available on NIST's GITHUB site.

## 2 Overview of Fused Filament Fabrication 3-D Printer

**Fig. 1:**
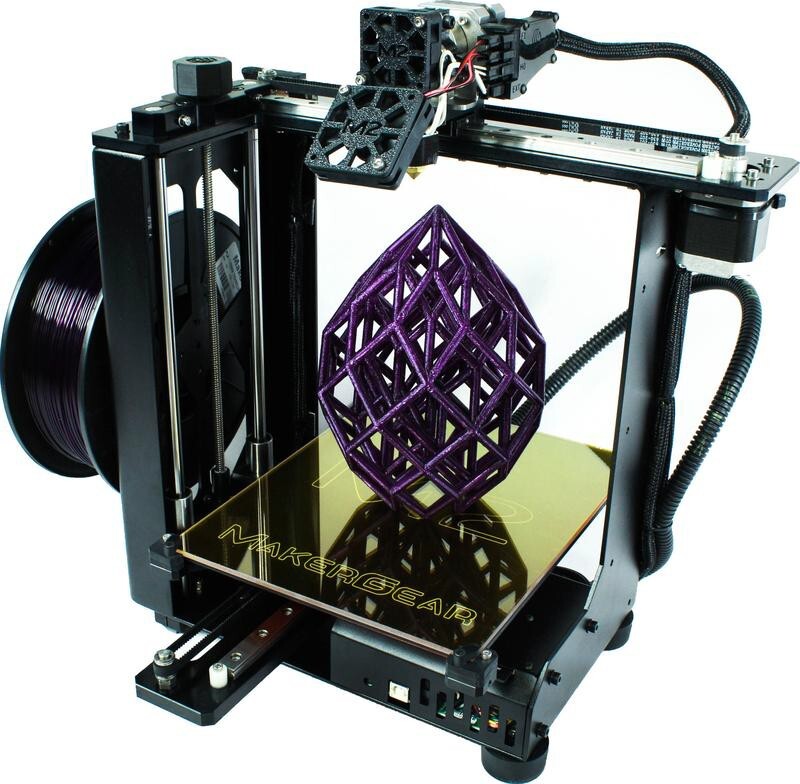
MakerGear M2 FFF 3D printer showing the single linear axis of the printing head.

Additive manufacturing, or 3-D printing, is an automated manufacturing process that deposits materials in a layer-by-layer fashion to create the final component. Creating a 3-D printed structure requires 3-D printing hardware. This hardware may take several forms, but it is often a three-axis gantry-style robot with an extrusion tool head. The operator programs this robot using G-code. These commands are interpreted by the firmware, which drives the stepper motors on the X, Y, and Z axes, and the filament extrusion components (E axis). Commands given to FFF 3-D printers of the type in Fig. 1 are based on International Organization for Standardization (ISO) 6983-1:2009 machine code format, commonly referred to as G-code [10]. The modifications to the ISO standard G-code are detailed in Ref. [11] and follow the interpreter outlined in Ref. [12].

The FFF selected for modification was the MakerGear M2 rev E and it is depicted in Fig. 1. This particular model was chosen because the hardware and software are open-sourced, allowing full control over the printer settings and operation. Furthermore, the MakerGear M2 is constructed such that the nozzle moves in only one linear direction; motion in orthogonal directions is achieved by moving the print table. This simplifes the paste tubing routing and reduces the length of tubing required to connect the nozzle to the pump.

**Fig. 2:**
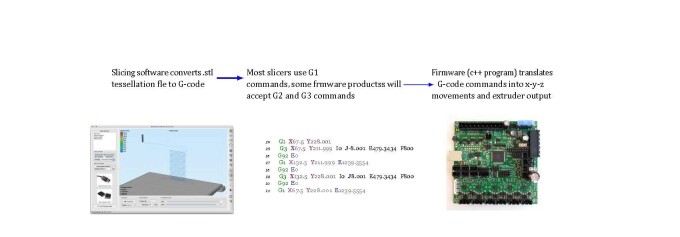
Schematic diagram of the 3-D printing process. Solid models generated in CAD software are converted to series of G-code commands which are interpreted by the firmware. G1 commands are interpreted as linear movements while G2 and G3 commands are interpreted as curvilinear movements in the clockwise or counter-clockwise direction, respectively.

The 3-D printing operation workflow can be described in three steps and is depicted in Fig. 2. First, a solid model is generated in a computer-aided design (CAD) software package, and it is typically stored in a common file format such as stereolithography (.stl), OBJ (.obj), or 3D Manufacturing format (.3mf) [13]. In this process, the solid model is broken down (tessellated) into a series of vertices and facet connections; although software can represent these objects using arbitrary polygons, in practice, they are almost always represented entirely by triangles. In the next step, the slicing software converts the tessellated representation into a series of robot movements expressed as G-code commands; the "slicing" refers to the layered nature by which extrusion printers operate. This step requires information about the printing process, such as: extrusion volume rate, nozzle speed, and materials properties. Information regarding the printing hardware can be entered through a user interface and used to compute printing speeds (for controlling the relative motion of the nozzle) and volumetric rate of deposited material (for controlling the paste pump). In the next step, the resulting G-code commands are sent to the printer and are executed sequentially.

The algorithms responsible for generating the G-code assume that the printing material is in filament form and extruded through a heated nozzle. In cement paste 3-D printing, the material is contained in a hopper in a fluid state and then it is pumped to a nozzle, from which it is deposited on the build plate. Starting and stopping commands (which are frequently used when printing with plastic) are challenging to execute with cement paste because the material has a yield stress. The material will not flow until the pressures in the pumping system exceed a threshold value. These important differences between plastic and cement paste extrusion physics are not accounted for during the "slicing" step (converting tessellations into G-code). Current slicing algorithms are not optimized to minimize starting and stopping of the extrusion axis. Once approach to overcome this challenge is to create the G-code commands without the use of a slicer. Using this approach, starting and stopping commands can be minimized, but the complexity of the printed structures is limited to the number of G-code commands that the user is willing to generate.

**Fig. 3:**
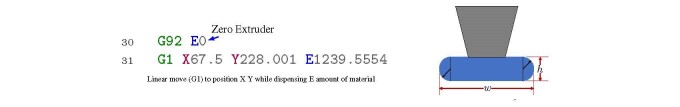
An alternative to computer-generated G-code is to write G-code manually.

The 3-D printer hardware is controlled by a single-board microcontroller, such as the one described in Ref. [14]. The microcontroller contains the built-in peripherals for controlling stepper motors and controlling a heated nozzle and build plate temperature. The G-code that is generated by a slicer becomes the input commands for the 3-D printer. The firmware loaded onto the microcontroller interprets the G-code commands and calculates the stepper motor movements [15]. Fig. 3 shows the G-code required to print material along a straight line. The first G-code command (line 30) stops the extruder and sets the current position of the extrusion axis to zero. The following extrusion commands will move the extrusion axis from this point over the number of specified units following the **E** command.^2^2The units of the extrusion axis movement will depend upon the calibration of the axis detailed in Sec. 4.
The second G-code command (line 31) provides the destination location (X and Y) and the volume of material to extrude (E). The volume is calculated using the cross-section of the filament and the path length. The filament cross section is computed using Eq. (1),

*A_f_* = (*π/*4 *-* 1) *h*^2^ + *wh* (1)

where, *h*, is the filament height, and *w*, is the filament width. The path length is the distance traveled from the current location to the desired destination. Eq. (1) assumes that the flament cross-section geometry is rectangular with semicircular ends.

**Fig. 4:**
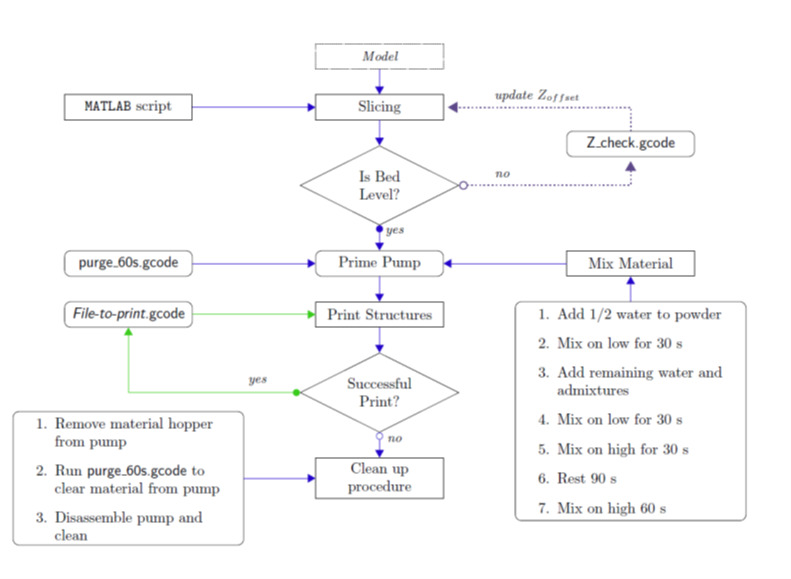
Flow chart describing the operation of the cement paste 3-D printer

The cement paste 3-D printer operation is summarized by the flow chart depicted in Fig. 4. The printing operation begins by choosing a model to print. This model may be generated using CAD software, or it may be a set of printer movements designed to probe a performance metric of the printer. The model is then fed to a slicer to generate G-code. If a CAD software package generated the model, slicing software may be used after the appropriate inputs have been determined. The paste printer is designed for printability studies investigating the material performance given a set of printing parameters. A set of MATLAB scripts (detailed in Sec. 2.2.1) was generated to simplify the slicing procedure for the models used for printability studies.The MATLAB code accepts user inputs such as the filament geometry, print speed, and location of the model on the build plate. It generates a set of G-code commands that minimize the starting and stopping of the extrusion axis.

The bed must be leveled relative to the nozzle to ensure consistent and repeatable deposition of material. Bed leveling is accomplished using the Z check.gcode function, which allows the user to determine the gap between the bed and the nozzle. After the bed is level to the nozzle, and the G-code has been generated, the paste is formulated and prepared. As soon as the paste is ready, the purge 60s.gcode function runs

the extrusion axis for 60 s to prime the extrusion pump and nozzle. Printing the structures can commence after the pump is primed. Printing continues until the paste rheology has changed to a point where a free-standing structure is not possible, or the stepper motor cannot drive the pump because the material has become too stiff. This point is the end of the printability window, and the cleanup procedure begins.

## 2.1 Hardware

**Fig. 5:**
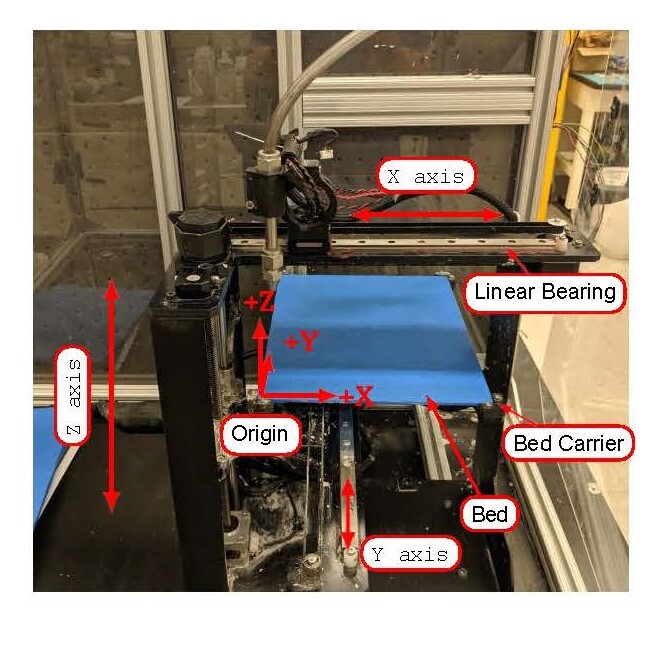
Overview of the polymer FFF 3-D printer converted to a cement paste printer.

The paste printer was built on a Cartesian 3-D printer platform, shown in Fig. 5. This printer is a gantry-style printer with three degrees of motion and two axes dedicated to material extrusion. The nozzle and bed on the X and Y axes, respectively, are mounted to a linear bearing to guide the movement. Each axis is controlled with a stepper motor and a timing belt drive train. In this particular 3-D printer model, the X and Y axes are mounted on different planes of motion. The X axis moves left and right, which minimizes the tubing movement connecting the nozzle to the pump. The Z axis platform is screw-driven and contains the Y axis linear bearing.

The printing bed is secured by the bed carrier at its four corners. The bed is a poly(methyl methacrylate) sheet cut to ft in the bed carrier. Adhesive tape covers the surface to improve the adhesion of the cement paste to the bed. Homing the axes defines the origin in the printer firmware and sets the coordinate systems for the G-code position arguments. The origin location is set by the end stops on the X, Y, and Z axes. The extrusion axis is set to zero and its current position becomes the origin.

## 2.2 Software

**Fig. 6:**
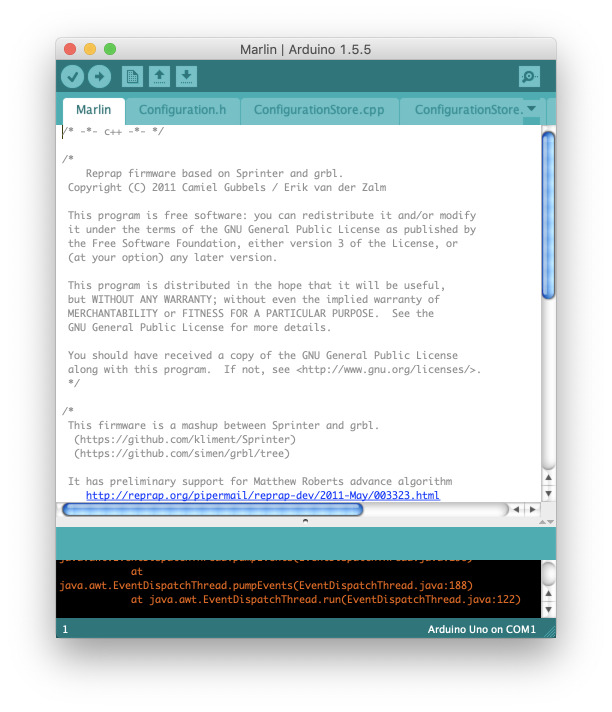
Marlin.pde opened in Arduino IDE version 1.5.5

The 3-D printer pictured in Fig. 1 was configured from the factory for a single material extrusion axis. A dual-material extrusion option is available, which can be used for the purpose of cement paste printing, although the modifications to the hardware may vary. The electronics hardware can accept a second material extruder; i.e., the RAMBo (RepRap Arduino-compatible Mother Board) board contains a second stepper driver. Printing with cement paste requires using this second extrusion axis if the original polymer material deposition axis is to be preserved. The dual extruder firmware must be installed To utilize the second stepper driver (if not installed from the factory). The dual extrusion firmware may be found here: http://frmware.makergear.com/#1. It is this version of the firmware that must be modified, as described in Sec. 3.2. Once the modifications are completed, the firmware is loaded onto the RAMBo board using the Arduino IDE (version 1.5.5). After the software modifications are complete, the file Marlin.pde is opened, as shown in Fig. 6, and compile.

Marlin is the firmware that controls the printer movements. It accepts G-code commands as inputs and converts them to step commands for the stepper drivers of the X, Y, Z, and E axes of the 3-D printer. Converting a polymer FFF printer to one that can dispense cement paste involves adding the hardware capable of handling the new material (pump, nozzle, etc.) and updating the firmware to accept these changes. The update to the firmware allows one to convert the number of stepper motor steps to the volume of material extruded by the pumping system. With this capability, the firmware is material agnostic and can be used without further modification.

## 2.2.1 Generating G-Code with MATLAB

**Fig. 7:**
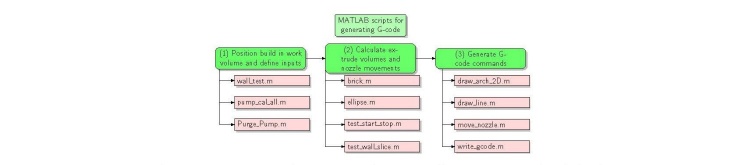
Workflow of MATLAB scripts used to generate G-code files, grouped by (1) initialization, where the build is positioned in work volume and inputs are defined, (2) geometrical calculations, where the extruded volume and nozzle movements are calculated, and (3) G-code generation, where G-code commands are generated.

Printing structures requires a G-code command for every movement of the printer. A package of MATLAB m-files has been created to facilitate the calculation of extruded volumes and nozzle positions for the wall structure, the pump calibration, and the pump purge routines. The advantage of these scripts is that they have parameterized the filament geometry, print speed, and location on the build platform. This allows users to vary multiple parameters and generate the corresponding G-code without recalculating X, Y, Z, and E for each step. The package is divided into three modules, shown schematically in Fig. 7.

The m-files in module 1 are used to define the input parameters for the print operation. The primary print inputs are the location on the build plate, the filament dimensions (height and width), and the pre-determined Z offset value. The G-code commands are stored in a cell array that is passed to the function write gcode, which loops through the cell array writing each cell to a line in the file. The MATLAB module generates G-code commands in two ways. Commands may be manually generated and stored in a cell array, as is done to create the header, preprint, and endprint cell arrays, or they can be generated automatically by specifying the points in the build volume and a filament geometry. Generating points automatically was the method used to generate the G-code for the printed structure in this study.

**Figure:**
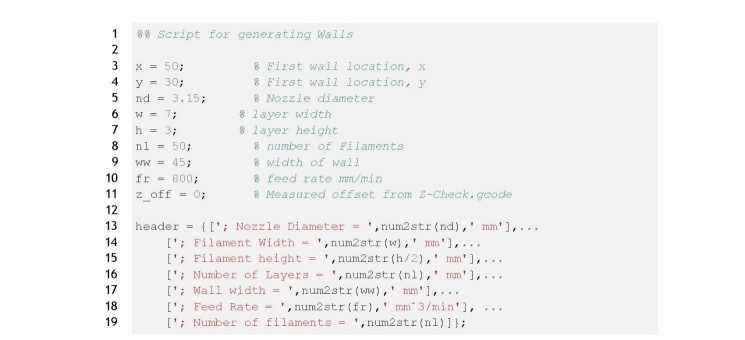
**Listing 1. **wall test.m: Input parameters for single filament stacking test.

The input parameters for the single filament stacking test (wall test.m) are shown in lines 2 through 11 of Listing 1; dimension values have units of millimeters. Lines 3 and 4 define the x-y position of the center of the wall, line 5 defines the nozzle diameter, and lines 6 and 7 define the filament width and height, respectively. Here, the filament cross section geometry is assumed to be rectangular with semi-circular ends. The flow rate is adjusted independently of the nozzle cross section and can be set to achieve the desired filament width. The wall structure requires the number of filaments and the width of the wall (lines 8 and 9). Finally, the speed of the print and the offset value is defined on lines 10 and 11. These input parameters are stored as variables to be used throughout the script and are prepended to the resulting G-code file.

G-code commands are required prior to executing a printing operation. These commands tell the firmware to operate in absolute coordinate mode, home the axes and select the appropriate tool. Lines 32 through 44 in Listing 2 show these commands, which are stored in the variable preprint.

**Figure:**
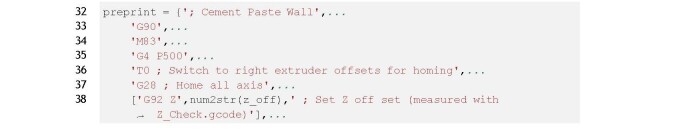

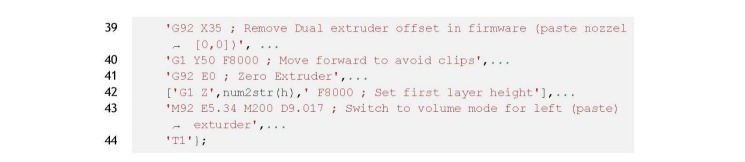
**Listing 2. **wall test.m: G-code commands to execute prior to printing operation.

Line 33 tells the firmware to operate the X-Y-Z coordinates in absolute mode, and line 34 sets the extrude to operate in relative coordinates. The measured offset value is applied to the z-axis in line 38. Line 43 updates the firmware with the calculated steps per unit volume for the paste extruder (**E5.34**), and sets the filament diameter to the internal diameter of the pipe (**D9.017**).

Lines 47 to 54, shown in Listing 3, are commands executed after the print is complete. These commands move the nozzle up 10 mm in the Z direction, line 51, and home the X-axis, line 53.

**Figure:**
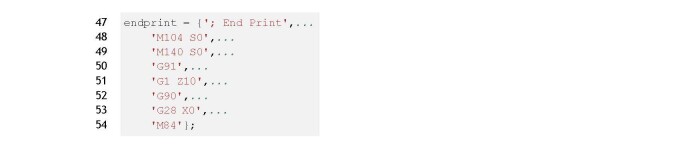
**Listing 3. **wall test.m: G-code commands to execute after printing operation.

The G-code commands for the filament stacking test are generated in the function test wall slice on line 58 of Listing 4. This function returns the commands to the cell array wall. Lines 60 through 66 create a file in the specified directory, and write the G-code commands to that file using the write gcode function.

**Figure:**
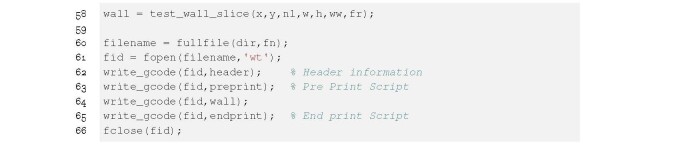
**Listing 4. ** wall test.m: Writing G-code to a fle from MATLAB.

The function test wall slice.m is called on line 58 of Listing 4 and is shown in Listing 5. It accepts seven arguments, which are used to calculate the X, Y, Z, and E values. Arguments x and y define the X and Y location of the center of the test structure on the build plate. Argument n1 defines the number of layers; w and h define the width and height of the filament, respectively; ww and fr define the width of the test structure and flow rate of the material. Here. test wall slice.m uses these arguments and, by assuming the geometry of the filament, calculates X, Y, Z, and E for each movement of the nozzle assuming the firmware is interpreting G-code in absolute coordinates.

A G-code command requires a location, a volume of extruded material, and the rate at which the material is extruded. The location, which consists of the X, Y, and Zvalues, is calculated from the user-supplied x and y arguments and the desired geometry (in this case, a single filament). The Evalue is the volume of the extruded material and is calculated by multiplying the cross-sectional area of the flament, *A_f_* by the path length, *L_p_*. *A_f_* is calculated by assuming a filament cross-section geometry. Here, the geometry was assumed to be a rectangle with circular ends as shown in Fig. 3. *L_p_* is the travel length from the current position of the nozzle to the desired position.

**Figure:**
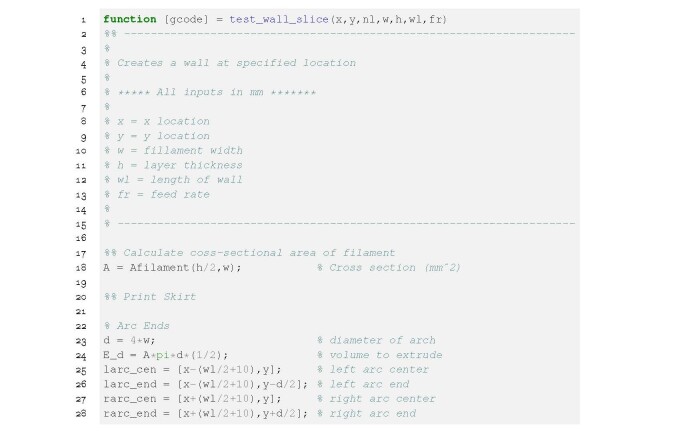

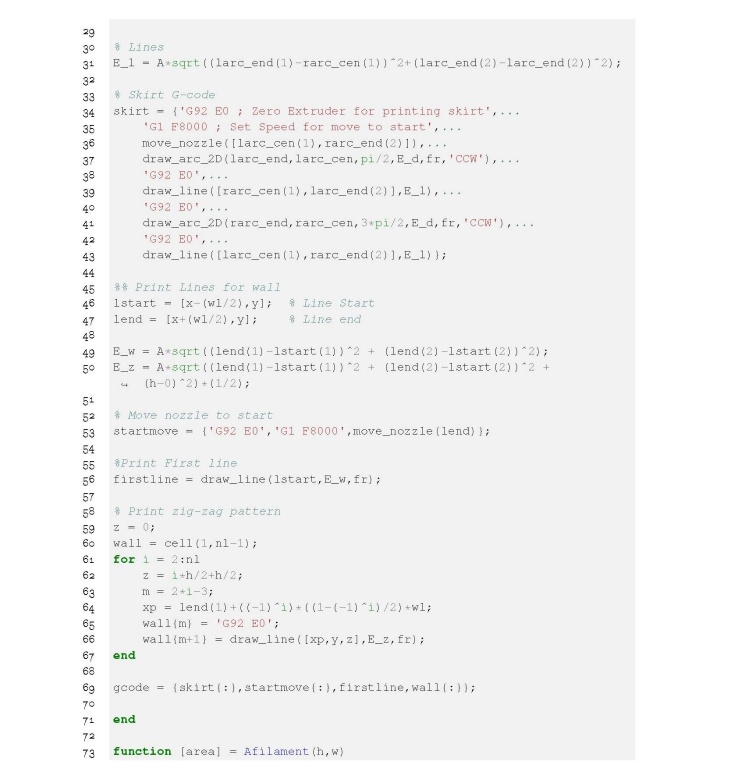

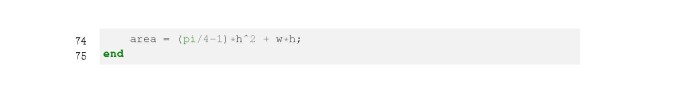
**Listing 5.** wall test slice.m: Calculating X, Y, Z, and E values for the filament stacking test.

## 2.3 G-Code Description

The paste printer was programmed using ISO 6983-1:2009 machine code format, commonly referred to as G-code [10]. To accommodate the paste and polymer nozzle, the firmware assumes the G-code follows the modifications to the ISO standard G-code that are detailed Ref. [11] and the interpreter outlined Ref. [12].

Listing 6 details common G-code commands used when programming the paste printer. Lines 1 to 8 are commands supplied to the printer prior to starting the print. T0 and T1 are the tool change commands for tool 0 and tool 1, respectively; the cement paste nozzle is tool 0 and the polymer nozzle is tool 1. Absolute positioning is set on line 4 with the G90 command. When this command is passed to the printer, the home position (determined from G28) becomes the origin in the build volume. All X, Y, and Z numbers are interpreted to be coordinates with respect to the origin.

**Figure:**
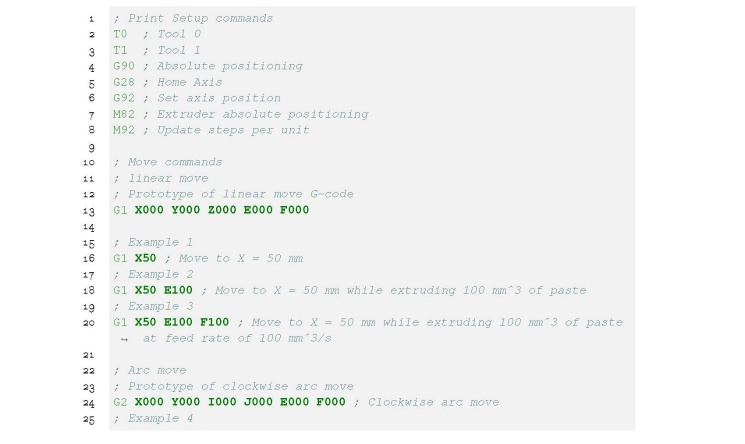

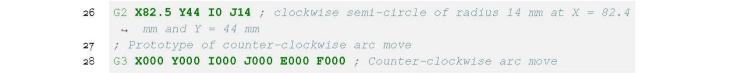
**Listing 6.** Common G-code commands used to program the paste printer..

The G92 command on line 6 moves an axis to a specified position from the origin. For example, an offset to the Z axis may be applied with the command, G92 **Z1**. This will move the Z axis 1 mm in the positive direction from the origin and redefine the position to be the origin. M82 on line 7 sets the extruder axis to absolute position mode. When the axes are homed using G28, the current position of the extrusion axis is set to zero. The extrusion axis may also be zeroed independent of the other axes by the command, G92 **E0**. Here, the current position of the extrusion axis is set at zero. Updates to the firmware configuration.h file are possible using M commands. For example, the steps per unit of an axis may be updated using M92. The extrusion axis steps per unit may be updated using the command, M92 **E000**, where **000** is the steps per unit value for the current tool.

To generalize the use of 3-D printers, and to simplify the configuration, all axes have a scaling parameter that is referred to as steps per unit. For the X, Y, and Z motion axes, the implied quantity is steps per linear dimension (steps/mm). For the extrusion axes, the implied quantity is steps per volume extruded (steps/mm^3^).

Linear moves are programmed using the G1 command, which accepts parameters **X**, **Y**, **Z**, **E**, and **F**. The position coordinates, **X**, **Y**, and **Z**, are with respect to the origin. **E** is the volume of material to extrude during the move and **F**, is the rate at which the material is extruded. Lines 13 through 20 are examples of using the G1 command. When the command is passed to the printer, the nozzle is moved from the current location to the specified location. If **E** and **F** are called, the printer will extrude the material at the feed rate during the movement.

Arc moves are possible using G2 and G3, as shown on lines 22 to 28. The syntax for arc command is similar to linear commands with the addition of parameters **I** and **J**, which specify the offset from the center of the arc that must be maintained during the move.

## 3 3-D Printer Modifications

Conversion of a polymer-based FFF printer to a cement paste 3-D printer requires both hardware and software modifications. The hardware modifications include a pump to control the deposition of the paste, a nozzle to control the geometry of the filament, and a hopper to hold the material during the printing operation. Software modifications involve updating header file definitions to account for the new hardware - stepper motor and gear box - and to disable software interlocks that would otherwise prevent printing cement paste at ambient temperatures.

## 3.1 Hardware Modification

The paste printer was designed to operate in a manner similar to a polymer printing operation. Movements of the nozzle are controlled by G-code commands that are given to the printer through the use of commercial software package compatible with the printer. Th e paste printing operation begins by mixing cement paste per the user's desired specifications. The mixed material is placed into the hopper. A purge protocol is executed until paste is extruded from the nozzle. Pre-prepared G-code files are executed to create the desired structure. The printed structure may be printed as many times as allowed by the kinetics of cement hydration and the limitations of the stepper motor and pump.

**Fig. 8:**
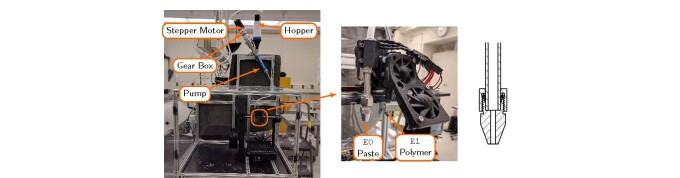
Overview of completed cement paste 3-D printer: (left) a view of the table, nozzle, and pump; (middle) a close up of the paste nozzle (E0) and polymer nozzle (E1); (right) cross section of paste nozzle.

The completed paste printer is shown in Fig. 8. Hardware modifications include a pumping system, a nozzle, and an external control system to drive the stirring mechanism. The left image shows the pump and hopper system mounted above the printer. Clear polyvinyl chloride (PVC) tubing, with an outer diameter of 9.5 mm and a wall thickness of 1.5 mm, connects the output of the pump to the nozzle. The tubing should be flexible to accommodate the X-axis movement of the nozzle and able to withstand the pressures generated when pumping cement paste. The tubing used in this application had a manufacturer-specified Shore A hardness of 65 with a 25 mm bend radius. The middle image in Fig. 8 shows the paste nozzle (E0 extrusion axis) and the polymer nozzle (E1 extrusion axis). A cross section of the paste nozzle geometry is shown on the right image of Fig. 8.

## 3.1.1 Pumping System

**Fig. 9:**
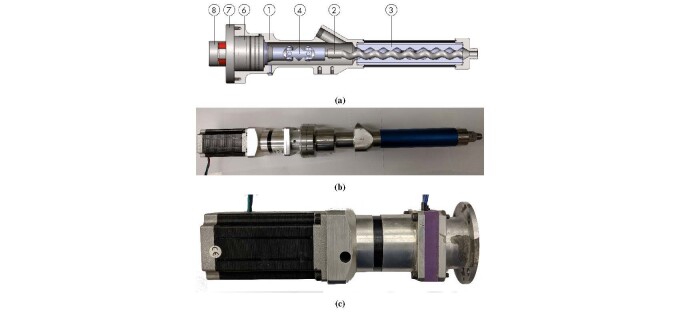
(a) Schematic diagram of rotor-stator cavity pump and (b) pump with stepper motor and gearbox attached. (c) Stepper motor and gear box with capacitive incremental encoder mounted to the end of the gear box.

The rotor-stator progressive cavity pump in Fig. 9 was used to supply cement paste to the nozzle. This type of pump was used because it is a low-shearing, positive displacement pump. The shear rates generated in this style of pump are considerably lower than gear-driven positive displacement pumps and may have a lesser influence on hydration kinetics.^3^3Exact shear rates for various styles of pumps are manufacturer specific. Consult manufacturer's documentation for the exact details of the pump. A positive displacement operates by trapping the fluid in a void between the rotor and the stator. As the rotor turns, the fluid is moved from the inlet to the outlet of the pump. This produces a flow that is proportional to the rotational speed of the pump and is independent of the pump outlet pressure.

A cross section of the rotor-stator progressive cavity pump is shown in Fig. 9a. Number 3 identifies the rotor and the stator, 2 is the pump inlet, and 4 is the U-joint coupling to the rotor. The U-joint is necessary because the rotor is eccentric to the stator central axis. This produces the void that moves the material through the pump. Number 8 is the connection to the stepper motor.

Fig. 9b is the complete pumping assembly. The stepper motor is attached to a 5:1 planetary gear box that is attached to the end of the pump at Number 8. The stepper motor is powered by a 2 A stepper motor driver (located on the RAMBo board) and has a zero-speed holding torque of 2 Nm. The 5:1 gear box is attached to the end of the stepper motor to increase the maximum torque applied to the pump. While the gear box increases the maximum torque, it also increases the rotation speed of the stepper motor, which is required to achieve the same pump flow rate. The maximum torque produced by a stepper motor is the zero-speed

holding torque. As the speed of the stepper motor increase, the torque decreases. Adding a gear box increases the torque by a factor of 5. Fig. 9c shows the stepper motor and gear box with an encoder. The encoder is used to monitor the speed of the pump and to calculate the flow rate of the material at the nozzle.

**Fig. 10:**
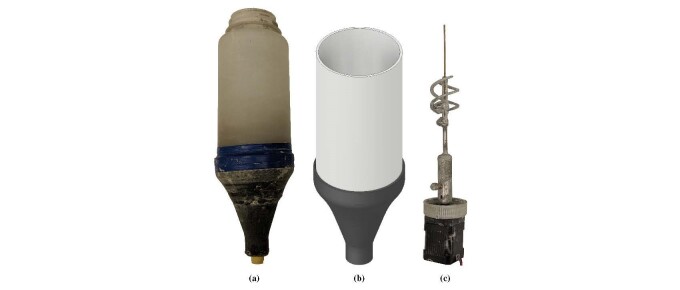
Hopper for cement paste system. (a) The hopper is a high density polyethylene container attached to a 3-D printed nozzle. (b) A CAD rendering of the hopper. (c) The stirring mechanism.

The hopper shown in Fig. 10 was used to supply the pump with material. The hopper is a high density polyethylene container with a 3-D printed funnel, shown in Figs. 10a and 10b. The funnel is used to help guide the material into the pump inlet, which is approximately 12 mm in diameter. The yield stress prevents the cement paste from flowing into the pump under its own self mass. To aid in getting the material to flow into the pump inlet, a stirring system is used to provide shearing stresses to the material; the cement paste is a shear thinning material. The stirring system, Fig. 10c, consist of a double helical rotor that is powered by a stepper motor and controlled through the external control system described in Sec. 3.1.3.

## 3.1.2 Nozzle System

The paste printer is capable of operating in two modes, single-material printing or dual-material printing. Either the cement paste tool or the polymer tool may be used in single material printing. It is recommended that the polymer tool be removed from the printer, and to print with cement paste in the single-material configuration. This is to prevent the polymer tool from interfering with the cement paste print.

**Fig. 11:**
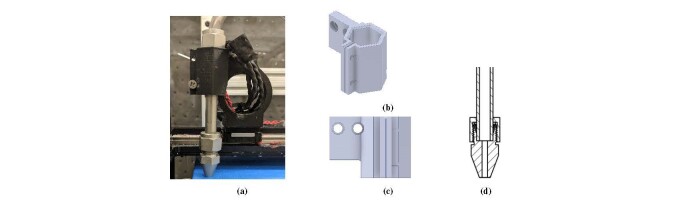
Nozzle setup in single-material printing configuration. (a) Paste nozzle in holder. (b) and (c) CAD representation of nozzle holder. (d) Cross section of paste nozzle.

Fig. 11 shows the paste tool in the single material, paste printing configuration. The paste tool consists of the paste nozzle holder (Figs. 11b and 11c) and the paste nozzle (Fig. 11d). The paste nozzle holder is attached to the side of the polymer tool, utilizing the existing hardware to secure the nozzle. The nozzle holder may be created using polymer FFF, as was done with the paste tool in Fig. 11a. The nozzle holder

utilizes screws to secure the paste nozzle in the holder. The paste nozzle and nozzle holder configuration allow for Z-axis adjustments of the nozzle by moving the nozzle up and down in the holder. The paste nozzle should be secured in the nozzle holder and adjusted to make contact with the bed. Fine adjustments of the gap between the nozzle and the bed are described in Sec. 5.2.

The paste nozzle consists of a stainless steel tube with a 9.5 mm outer diameter and 1.5 mm wall thickness and a nozzle tip constructed from stainless steel. Fig. 11d shows a cross section of the paste nozzle. The nozzle was designed to be compatible with swage-type fittings. The exact design of the nozzle will depend on the application. There are, however, two design features to consider in Fig. 11d. The tubing mates to the nozzle at a 90° angle. The was done to replicate the geometry of a capillary rheometer. Compatibility with commercially-available swage-type fittings allows operators to fabricate nozzle geometries, as required by particular applications.

Operating the paste printer in dual material configuration requires both the paste and polymer tools depicted in Fig. 8. The paste tool is offset to the left of the polymer tool. The offset between the two tools is 25 mm along the X axis and must be accounted for when generating G-code. The paste and polymer tools can only be used sequentially. A tool change command must be passed to the printer in the G-code, followed by an update of steps per unit as described in Sec. 3.3.

## 3.1.3 External Control System

The pumping system, added to the extrusion axis, requires a stirring mechanism to maintain fow of the material in the hopper. The RAMBo board is equipped with fve stepper drivers for the X, Y, Z, E0, and E1 axes. An additional control system was required to drive the stirrer stepper motor; the stepper motor and stirrer assembly was attached to the hopper lid. The external control system (ECS) expands the number of stepper drivers available to the paste printing and provides additional input and outputs that can be configured to monitor printer performance, e.g., an encoder added to the pump to measure flow rate.

**Fig. 12:**
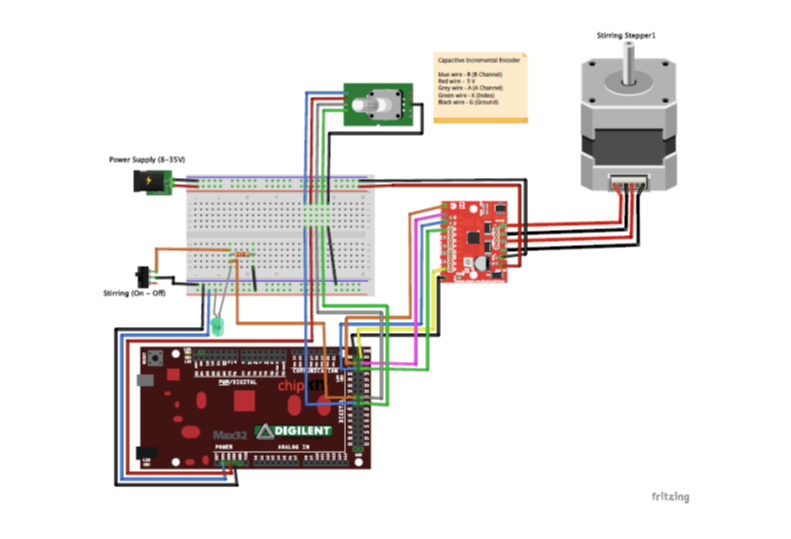
Stirring control system with capacitive incremental encoder hardware diagram. The capacitive incremental encoder is attached to the progressive cavity pump and is used to monitor the rotation of the pump. The encoder is calibrated to pump flow rate which is used to calibrate the extrusion axis. The stirring stepper motor is connected to a stepper motor driver which is controlled from the microcontroller.

A 32-bit PIC32 microcontroller is used for the ECS. It has the necessary number of inputs and outputs to control the stirrer stepper motor and accommodate future hardware such as the active mixing nozzle. A schematic diagram of the stirring control system is given in Fig. 12. A stepper motor driver based on the Allegro A4988 stepper driver chip was used to drive the stirring stepper motor. The logic to turn the stepper motor on and off is handled in the ECS firmware, which monitors a voltage signal from the switch. When the switch is in the "on" position (printing is active), the firmware makes a call to the AccelStepper library http://www.airspayce.com/mikem/arduino/AccelStepper/), which sends a step command to the stepper driver. This is repeated until the switch is put in the "off" position.

The ECS can monitor the fow rate of the extrusion axis using a capacitive incremental encoder (CIE).The encoder pulses are monitored using the encoder library found here: https://github.com/PaulStoffregen/Encoder. The encoder pulse can be calibrated to pump flow rate using the procedure in Sec. 4.

## 3.1.4 End Stop Modifications

The nozzle for the paste printer is located approximately 25 mm to the left of the polymer extrusion nozzle. The home position of the nozzle must be determined at the beginning of each print because it is the origin for the X, Y, and Z coordinates in the G-code. Homing the axis is accomplished by the G28 command, which moves the X, Y, and Z axes toward their end stops until they are triggered.

Modified Xand Yaxis end stops, shown in Fig. 13, are required to center the paste nozzle on the bottom-left corner of the build plate. The modified end stops extend the adjustable range of the end stops by the use of a M3 *✕* 10 mm screw. The extended length of the screw allows the operator to fine tune the end stop adjustment and will provide flexibility to accommodate other nozzle geometries and sizes.

## 3.2 Software Modifications

The MakerGear M2 Rev. E Marlin-variant firmware can be downloaded here: http://frmware.makergear.com/#1. To operate both the paste and polymer extrusion axes, the dual material variant must be used. The configuration of the Marlin firmware is set in the configuration.h

file. This file sets the parameters such as end stop positions, communication between the RAMBo board and the computer, temperature control, and stepper motor configuration. Three modifications must be made to configuration.h to allow the printer to operate as a cement paste printer.

Listing 7 shows the temperature lockout modifications required in configuration.h. The minimum allowable extrusion temperature is set on line l17 by defining a macro EXTRUDE_MINTEMP. If the bed or nozzle thermocouple measures a temperature below this value, the firmware pauses the print until the temperature is exceeded. Since cement paste is printed at ambient temperature, EXTRUDE_MINTEMP was set to 15 °C, below the ambient temperature at which the print would be executed.

**Figure:**
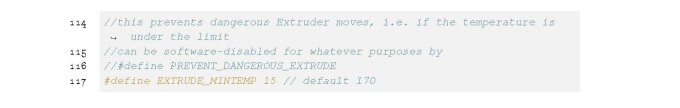
**Listing 7.** configuration.h: Allow stepper motors to move when temperature is below set point.

The stepper motor configurations in configuration.h are shown on line 175 of Listing 8. These values represent the calibrated steps per unit for the stepper motors, where the "unit" can either be a length or volume depending upon the calibration. The X, Y, and Zaxis stepper motors are calibrated to displacement (mm) and the extrusion axis, E, is calibrated to volume of material (mm^3^). The calibration values are entered as a vector in the macro DEFAULT_AXIS_STEPS_PER_UNIT. The components of the vector represent the X, Y, Z, and Eaxis steps per unit. The steps per unit for the extrusion axis is calculated in Sec. 4. The calibrated value is entered in the fourth component of the vector on line 175.

**Figure:**
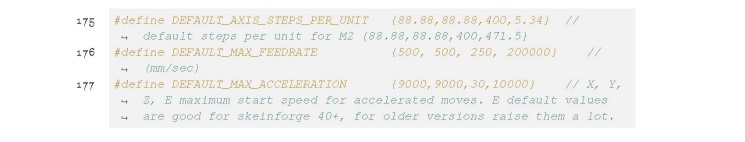
**Listing 8.** configuration.h: Modify the steps per unit for pump and gear box

The final modification to configuration.h is to increase the available current to the extrusion axis. Increasing the current applied to the stepper motor increases the maximum torque. The current is set by the E0_CURRENT macro on line 197 of Listing 9. The range of acceptable values is 0 to 255, where 0 is 0 A and 255 is 2 A.

**Figure:**
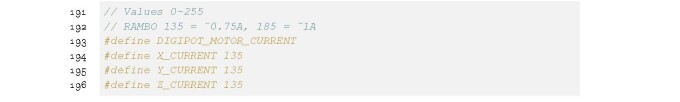

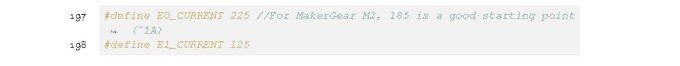
**Listing 9.** configuration.h: Increase the current supplied from stepper motor controller

## 3.3 Operating in Dual-Material Mode

The RAMBo board has two stepper motor drivers dedicated to material extrusion, defined as E0 and E1.

In a typical dual material extrusion application, the nozzles for both materials are identical and the same steps per unit value can be used for both extrusion axes. The steps per unit value, defined on line 175 of Listing 8, is applied to both extrusion axes. The firmware does not allow separate definitions of the steps per unit for E0 and E1. The paste printer is able to utilize both extrusion axes, where E0 is dedicated to cement paste deposition while E1 is the polymer extrusion nozzle. Because the firmware assumes both E0 and E1 are identical, the extrusion axes steps per unit must be updated before printing with the polymer or paste nozzle. This can be accomplished in the G-code using the tool change command, T0 or T1, and the M92 command.

T0 or T1 tells the frmware to switch to the E0 or E1 extrusion axis, and M92 followed by **Exxx** tells the frmware to update the extrusion axis steps per unit to the value xxx. An example of a tool change command from E0 to E1 is given in Listing 10.

**Figure:**
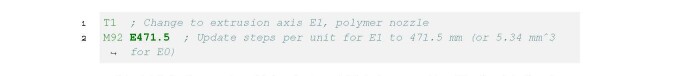
**Listing 10.** Tool change command from E0 to E1. The command to change tools is given on line 1, followed by an update of the steps per unit for the selected tool.

Line 1 tells the firmware to switch to extrusion axis E1 (polymer nozzle) and line 2 updates the steps per unit for this nozzle. Each time the tool change command is given, it must be followed by the command to update the steps per unit for the new nozzle.

## 4 Calibration of Cement Paste Pump

The software modifications outlined in Sec. 3.2 require the steps-per-unit volume for the extrusion axis (line 175 of Listing 8). The steps per unit values are calculated as the ratio of the steps per revolution of the pump to the volume of material extruded per one pump revolution. The stepper motor that is attached to the extrusion axis is a 200 step per revolution National Electrical Manufactures Association (NEMA) size 23 stepper motor, attached to a 5:1 gear box. The stepping motor is operated in micro-stepping mode, which allows the stepper motor to rotate 1/16 of a step per one step command given by the stepper driver. This reduces the minimum shaft rotation angle per step, which allows for more precise movements of the stepper motor. The volume extruded per pump revolution is determined by a bench top calibration of the extrusion axis pump, which is discussed in detail below. After completing this calibration for the components used here, the signal steps required per unit volume extruded is calculated using Eq. (2). The expressed uncertainty represents the 95% confidence interval about the mean value. It was calculated following the procedure outlined Ref. [16] where the uncertainty from the volume calibration is propagated to Eq. (2).

$S=\frac{\text{steps/rev}}{\text{volume/rev}}=\frac{\left(200\text{ steps/rev}\right)\left(16\right)\left(5/1\right)}{3.21\text{ mL/rev}\left(1000{\text{ mm}}^{3}/\text{mL}\right)}=\left(4.98\;0.62\right){\text{ steps mm}}^{-3}$(2)

**Fig. 14:**
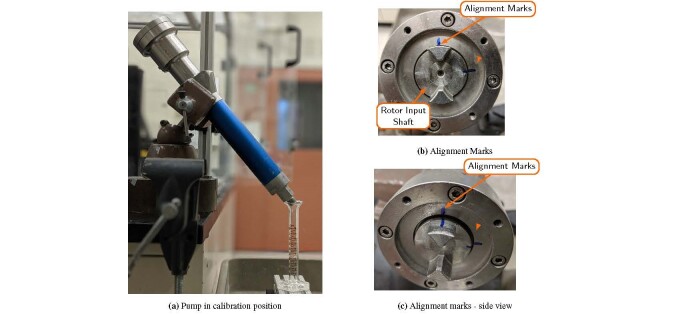
Extrusion pump in calibration position. (a) The pump is secured in a fixed location and a graduated cylinder is used to measure the volume of material dispensed from the pump. (b) and (c) Alignment marks are used to count the number of rotor rotations.

The extrusion axis pump (Fig. 9) was calibrated by mounting the pump to a fxed location and turning the pump manually, without the aide of the stepper motor or gear box, as shown in Fig. 14. A graduated cylinder is placed under the output of the pump to measure the discharge volume. The pump revolutions are calibrated to the discharge volume by measuring the volume of material dispensed into the graduated cylinder as a function of rotor revolutions. One rotor revolution is a 2*π* rotation of the rotor input shaft in the counter-clockwise direction. Rotor revolutions are counted using two alignment marks perpendicular to each other. The alignment marks should cross from the rotor input shaft to the housing as shown in Figs. 14b and 14c. The portion of the mark on the housing remains stationary as the rotor input shaft is turned. Revolutions are counted when the marks are aligned.

The extrusion axis pump was calibrated with a mineral oil, which exhibits Newtonian characteristics, and also with NIST SRM 2492, a Bingham fluid [9]. The calibration is the same for both fluids, but the yield stress (the propensity for forming voids in the extruded mass) and the opacity of SRM 2492 make direct volume measurements more challenging. The density of SRM 2492 was measured using a graduated cylinder and a balance, and then this value was used to compute the volume of extruded material from mass measurements. The procedure for calibrating the extrusion axis pump is listed below:

1. Secure pump in a fixed location. Add two alignment marks to the rotor input shaft and adjacent housing as shown in Fig. 14.
2. Place material collection container under pump output.
  • When using mineral oil, the collection container is a graduated cylinder with 0.2 mL precision.
  • When using SRM 2492, the collection container is a balance with 0.01 g precision.
3. Add calibration material to the pump inlet. The pump pictured in Fig. 9 will hold *≈*15 mL.
4. Prime the pump by rotating the rotor input shaft CCW until material is extruded from the pump outlet. Continue to fill the pump with the calibration material as needed.
5. To make a calibration measurement, rotate the rotor input shaft until material is extruded from the output.
6. Once material is observed, align the alignment marks as shown in Figs. 14b and 14c.
7. Rotate the rotor input shaft the desired number of rotations.
  • When using mineral oil, measure the extruded volume to the nearest demarcation.
  • When using SRM 2492, measure the mass to the nearest 0.01 g.
8. Repeat the measurement five times, while continuing to add calibration material to the pump, before proceeding to the next calibration point.
9. A total of four calibration points, e.g., 1, 2, 3, and 4 revolutions, are required to complete the calibration.

The calibration constant is calculated by plotting the volume of extruded material against the pump revolutions. Linear least squares regression is used to ft a model, Y = *β* X. Calibration with SRM 2492 relies on mass measurements that must be converted to volume. The density of SRM 2492 is estimated by five mass and volume measurement pairs. The volume is estimated from the ratio of the average of the measured mass to the material density. The uncertainty of the volume estimation was calculated following the procedures outlines Ref. [16].

**Fig. 15:**
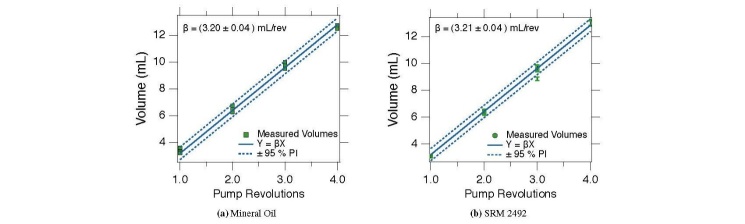
Calibration curve for rotor-stator cavity pump depicted in Fig. 9 using (a) a Newtonian fluid and (b) a yield stress fluid. The error bars represent the 95% confidence interval about the mean and the dashed lines are the 95% prediction interval estimated by linear least squares regression.

The calibration curves for both the mineral oil and SRM 2492 are shown in Fig. 15. The calibration constant for the mineral oil is estimated to be (3.20 ± 0.04) mLrev^-1^ and (3.21 ± 0.04) mLrev^-1^ for SRM 2492. Here, the uncertainty represents the 95% confidence interval about the mean value. The difference between these calibration constants is one-fourth of the uncertainty in either value, indicating that the calibration is insensitive to fluid properties.

## 5 Demonstration of the Printing Process

Operating the paste printer requires several steps, which are summarized in Fig. 2. Printing with cement paste follows many of the same steps followed in conventional FFF 3-D printing. First, a solid model is sliced to generate G-code. A software interface is used to pass the G-code file line-by-line to the printer firmware. The firmware interprets the commands and moves the printer accordingly. Cement paste printing follows this approach, but it introduces several significant differences that must be addressed to ensure a successful print.

## 5.1 Slicing

Slicing, or generating G-code, presents the first significant challenge to cement paste printing.Commercial and open source slicing software packages accept solid models (converted to an appropriate format) and output G-code commands. The challenge with using such slicers to generate G-code for cement paste printing is that the algorithms responsible for generating the G-coder operate as black boxes. The operator enters a set of input parameters and the G-code is generated automatically. It is difficult for the end user to customize the output of the slicers to limit the occurrence of commands that start and stop the extruder. The result is poor print quality due to movement of the printer, and not due to the material characteristics.

**Fig. 16:**
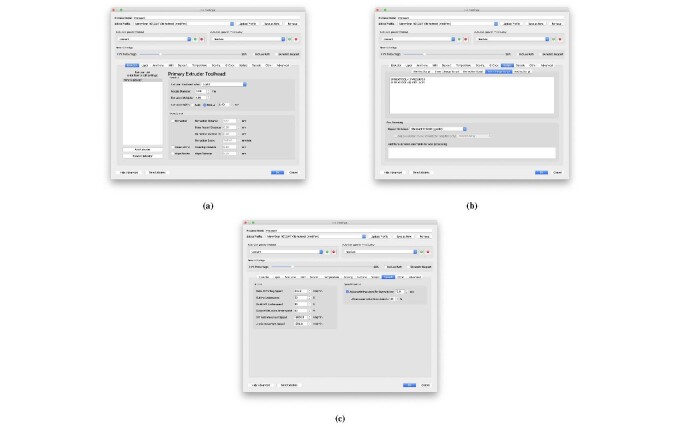
Slicing settings using Simplify3D. (a) Extruder options set filament width and height. (b) A tool change script is required to update steps per unit for the new tool. (c) Speed settings are set here.

Fig. 16 shows an example of initializing slicing settings for 3-D printing. There are many options available to the operator, but very few are relevant to cement paste. One approach is to disable the higher level features of the slicer such as supports, temperature, cooling, and retractions. These are specific to polymer FFF and can cause problems with cement paste 3-D printing. Fig. 16a shows window in the slicer where the nozzle geometry may be entered. Here, the nozzle diameter has been updated to the paste nozzle geometry. Fig. 16b shows a script that inserts the M92 command after the tool change command. This is required for dual material printing. Finally, Fig. 16c shows where the speed is updated.

When using commercial slicing software, the user is dependent upon the inherent slicing algorithm. For simple structures, following the aforementioned recommended settings when printing cement paste will increase the chances of a successful print. For more complex structures, however, it is almost certain that the built-in algorithm will insert commands that will not be suitable for paste printing.

Instead, operators are advised to generate G-code manually, particularly for simple geometric structures. Manual generation of G-code ensures full control of the printer, but it limits the printable geometries and can be a time consuming process. To aide in the generation of G-code for simple structures, MATLAB scripts were generated that allow operators to generate G-code for pre-defined structures. The function of the MATLAB scripts is described in Sec. 2.2.1. These scripts do not take an arbitrary object and generate G-code, rather, they act to standardize the printing parameters for pre-defined geometries and update the G-code accordingly. These parameters are the location of the object on the build platform, the size of the filament, the number of layers (where applicable), and the speed. The MATLAB scripts are capable of generating bricks, lines, walls, and ellipses.

**Fig. 17:**
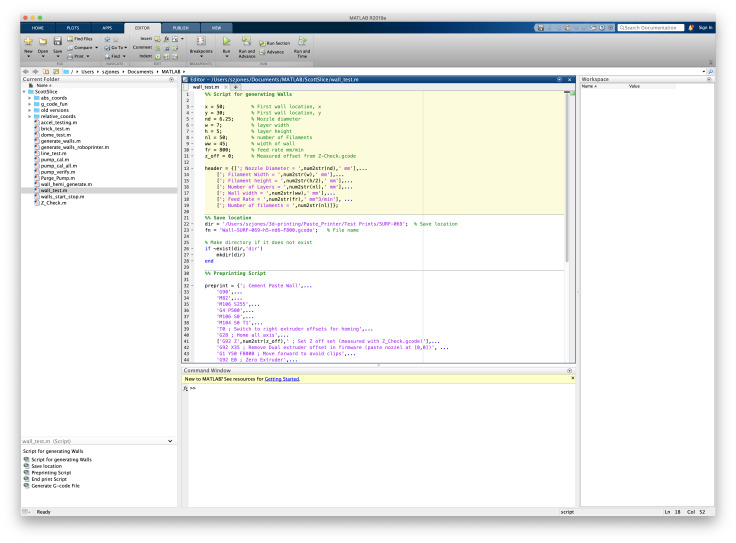
MATLAB interface for generating single-filament stacking test G-code.

To install the MATLAB scripts, place the folder containing the m-files in the MATLAB directory. Add the directory to the current path. The MATLAB interface is shown in Fig. 17. To generate the G-code, update the parameters as shown in Listing 1 and click "RUN". This will save the G-code in the specified directory.

## 5.2 Bed Leveling

**Fig. 18:**
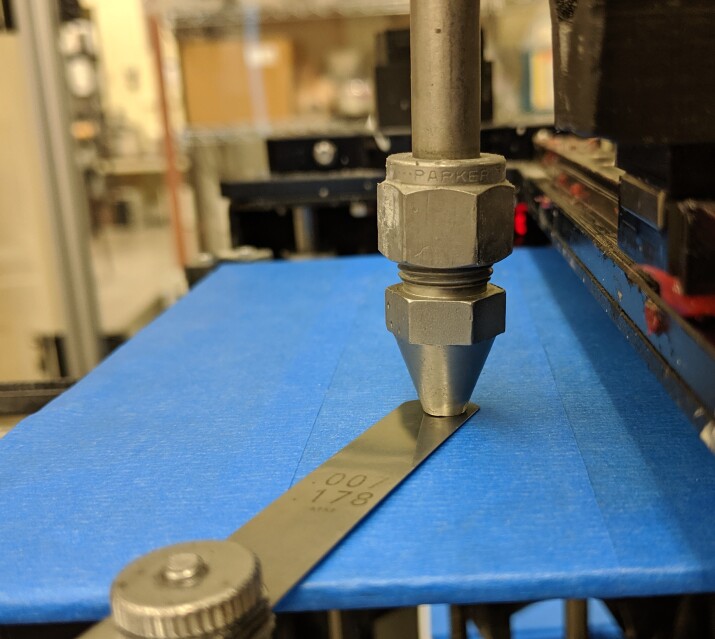
Using feeler gauge to measure the gap between the bed and the nozzle. Apply the measured offset to the G-code.

**Fig. 19:**
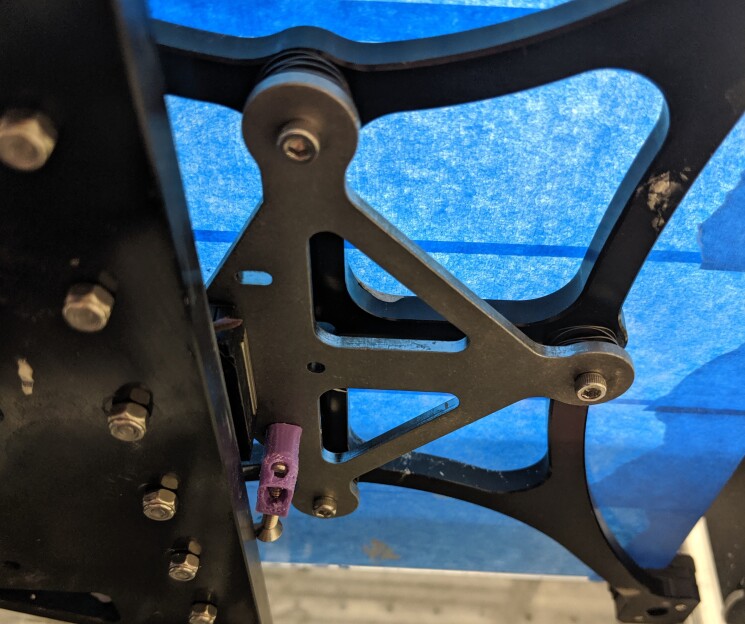
Adjustment knobs used to level bed.

 Leveling the bed is critical to ensuring that the deposited filaments have consistent size and shape throughout the print. To level the bed, execute the G-code Z offset.gcode shown in Listing 11. This function moves the nozzle to a point on the bed and pauses for 60 s. Using a feeler gauge, measure the gap between the nozzle and the bed as shown in Fig. 18. Repeat for at least three locations on the bed (by modifying Listing 11) and adjust the knobs shown in Fig. 19 until the gap is the same at all locations.

**Figure:**
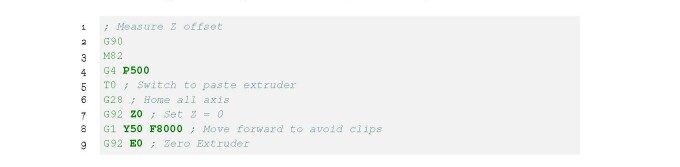

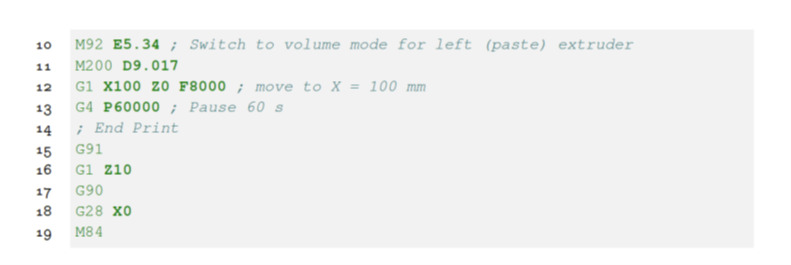
**Listing 11.** Z.offset.gcode: Moves nozzle to a point on the build platform and pauses for 60 s.

Once the gap is the same at three locations on the build plate, use the gap thickness to update the Z offset value and then generate the G-code. This will update the G92 **Z0** command with the measured gap. The newly generated G-code will incorporate the information, so that specifying a Z height of 0 will ensure there is no gap between the nozzle and the bed.

## 5.3 Purging the Pump 

After the G-code has been generated and the bed is level, the print is ready to begin. The operator should prepare the paste mixture per the requirements of the experiment. Fig. 7 suggests a mixing procedure, but it is not required for this printer. Place the material in the hopper after mixing is completed. Consolidate the material in the hopper using a rod with a diameter of approximately 5 mm. Be sure to push material from the hopper into the pump inlet. Install the stirring system and turn it on.

The pump is primed by running the E0 extrusion axis for 60 s using the G-code in Listing 12.

**Figure:**
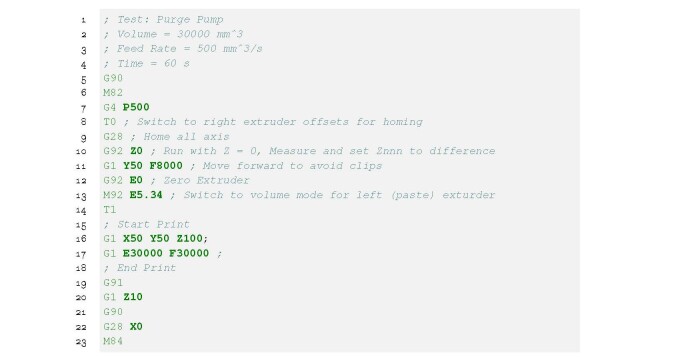
**Listing 12.** purge-60s.gcode: Moves nozzle to a point on the build platform and runs the pump for 60 s.1

**Fig. 20:**
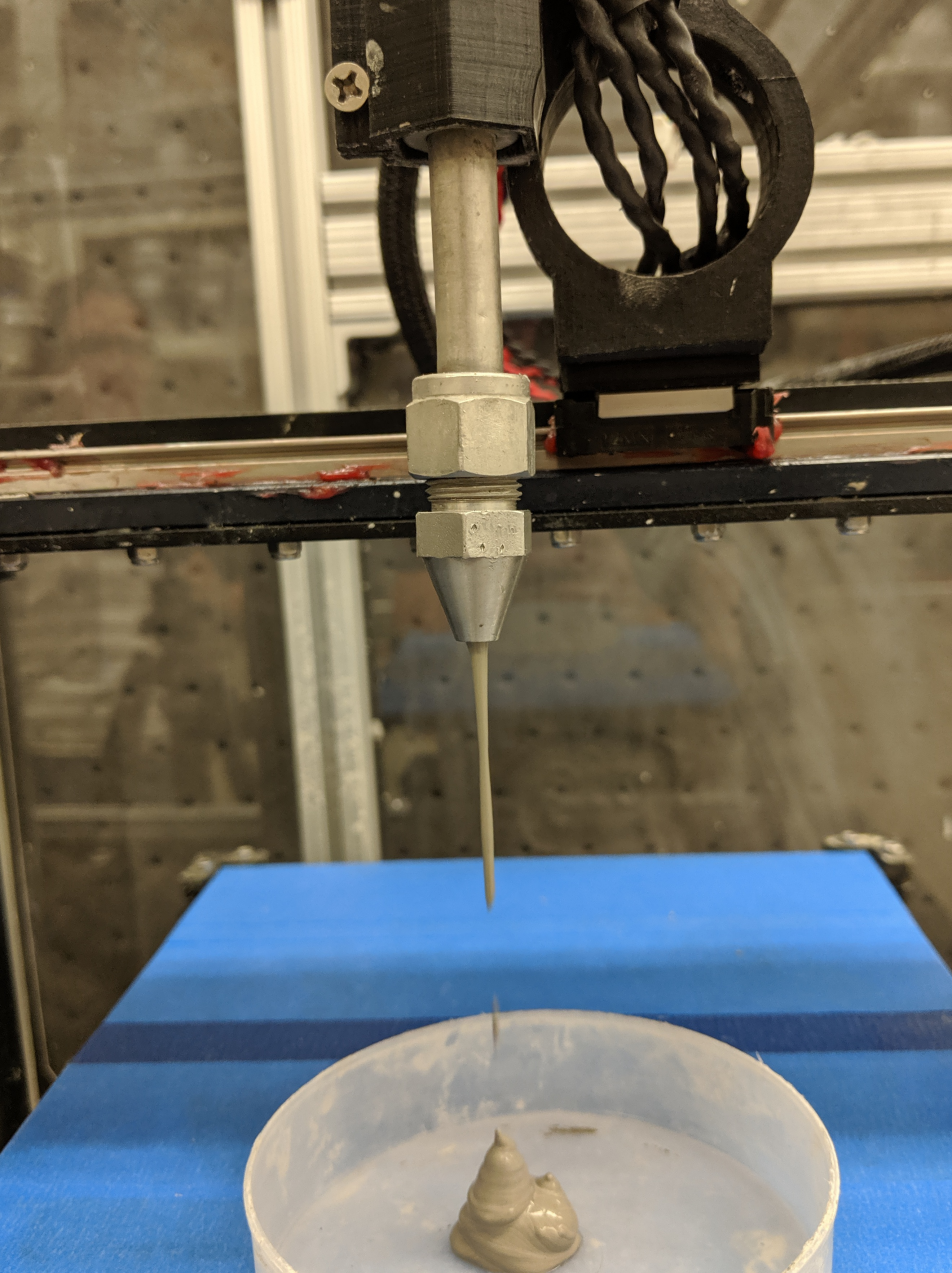
Purging the pumping system. The purge is complete when a steady fow of materials exits the nozzle.

Lines 5 to l3 home the axes and set the steps per unit. Line 16 moves the nozzle to a point in the build volume that will allow the operator to collect purged material and line 17 runs the pump for 60 s. Run the purge program as many times as required until the material exiting the nozzle appears as shown in Fig. 20. A consistently steady flow indicates the air has been purged from the system and the hopper is supplying material to the pump.

## 5.4 Printing Structures 

**Fig. 21:**
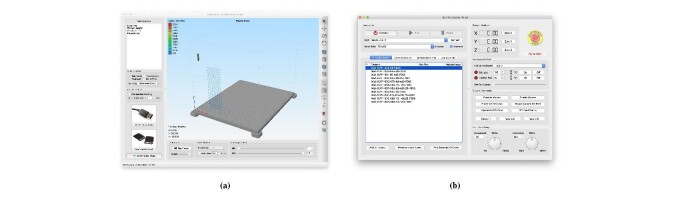
G-code files are passed to the printer using commercial or open-source software packages. Simplify3D can be used (a) to preview the print and (b) as a control pane to interface with the printer firmware.

G-code is passed to the printer through a software interface such as the one depicted in Fig. 21. The G-code may be opened and previewed in the software, as shown in Fig. 21a. If the code is deemed acceptable, the code can be passed to the printer, which will start the print.

G-code stored in a local directory may pass directly to the printer by selecting its location in the software panel shown in Fig. 21b. This feature is useful for running the same G-code repeatedly. This panel

also allows the operator to control certain features of the printer and provide an emergency stop feature that interrupts the G-code and stops the printing.

Using the Simplify3D software, a print is executed from the control panel. Load the G-code corresponding to the desired print into the software by clicking the "Add to Library" button in Fig. 21b. To run the G-code, select the file name and click "Run Selected G-code." The control panel will read the G-code and pass the commands to the printer line by line.

**Fig. 22:**
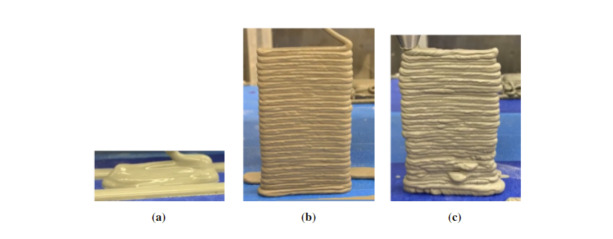
An example of prints that may be executed with the paste printer. This experiment was designed to test the number of layers a mixture can achieve as a function of hydration time. As hydration continues past (a) 30 min, (b) 60 min, and (c) 90 min, the quality of the printed structures increases and then decreases.

An example of a successful series of prints is given in Fig. 22. This experiment was designed to test the total number of layers a paste formulations can achieve as a function of the time after hydration. The material was mixed, added to the hopper, and the same G-code file was run at 15 min intervals.

Fig. 22 shows that the quality of the print, in terms of number of printed layers and overall adherence to the desired shape, changes with time. Early in the hydration process, the materials is not able to retain its shape. As hydration continues, more layers are possible until the quality begins to decrease. At this point, the material becomes difficult to pump and the end of the printing window has been reached.

## 5.5 End of Printing

The end of the printing is achieved when the quality of the print has reduced to a point determined by the operator or if pumping becomes difficult. Typical pumping difficulties include stalling of the stepper motor, large air voids, and blockages. At this point, the clean up process can begin.

Clean-up starts by removing the hopper from the pump. If the pump is able to move, i.e., no blockages or stalling, run the purge G-code to remove all material from the rotor and stator as well as the tubing and nozzle. Disassemble the pump and nozzle and clean thoroughly, removing all cement paste. After cleaning, apply mineral oil to the rotor and the stator to prevent cracking of the rubber stator.

## 6 Conclusion

A cement paste 3-D printer was assembled using a commercial fused filament plastic printer. The plastic printer was modified by attaching an extrusion cement paste nozzle as a second tool head and adding a cement paste delivery system consisting of a hopper, a stirrer, and a pump. The pump is controlled directly by the plastic printer firmware. The hopper stirrer was controlled by an external controller, that can be further modified to facilitate more complex operations and material formulations.

Software modifications include updating the configuration.h file in the printer firmware for cement paste pump and nozzle. The resulting firmware is compatible with existing commercial and open-source slicers. A set of MATLAB routines was written to simplify the slicing process for simple geometries relevant to cement paste additive manufacturing. The software modifications were discussed in detail and are made publicly available.

The resulting apparatus is capable of printing simple shapes, which can be used to characterize the printability of a material-process combination, as a function of material hydration time. Furthermore, the device can be used to identify and quantify the rheological and mechanical properties that result in successful builds.

## Supplemental Materials

Supplemental Materials may be found at https://github.com/usnistgov. Supplemental materials include the software to control the stirring stepper motor and CAD models to create components to complete the modification of the MakerGear M2 printer.
